# Female sexual dysfunction among foreign and Australian-born women: a cross-sectional study

**DOI:** 10.3389/frph.2026.1855626

**Published:** 2026-06-16

**Authors:** Negin Mirzaei Damabi, Mumtaz Begum, Jodie C. Avery, Salima Meherali, Zohra S. Lassi

**Affiliations:** 1 Robinson Research Institute, Adelaide University, Adelaide, SA, Australia; 2 School of Public Health, College of Health, Adelaide University, Adelaide, SA, Australia; 3 Adelaide Medical School, College of Health, Adelaide University, Adelaide, SA, Australia; 4 Faculty of Nursing, University of Alberta, Edmonton, AB, Canada

**Keywords:** Australia, equity, migration, sexual function, women

## Abstract

**Background:**

Female sexual dysfunction (FSD) affects a substantial proportion of reproductive-aged women (15–49 years) globally, yet little is known about how migration shapes sexual function. This study compares and describes the patterns of FSD among foreign-born and Australian-born women residing in Australia.

**Methods:**

This cross-sectional study recruited women through an online survey using a convenience sampling approach with equal recruitment targets for foreign-born and Australian-born women. FSD was assessed using the validated Female Sexual Function Index (FSFI), with dysfunction defined as a total score of 26.55 or below. Patterns of FSD were compared between groups and stratified by sociodemographic characteristics. Multivariable logistic regression was performed to compare FSD among overseas-born women with Australian born women, controlling for age, education, marital status, religion, area-level socioeconomic position, and gravidity.

**Results:**

A total of 678 women were included in the final analysis (342 Australian-born, 336 foreign-born). About 47.1% (95% CI: 41.7–52.5) Australian-born and 46.1% (95% CI: 40.7–51.6) foreign-born participants had FSD. Mean FSFI total scores were nearly identical (Australian-born: 26.35 ± 5.72, 95% CI: 25.74–26.95; foreign-born: 26.41 ± 5.69, 95% CI: 25.80–27.03; *p* = 0.876), with both groups falling just below the dysfunction threshold. Scores across all six FSFI domains were broadly comparable. There were substantial differences in the distribution of FSD cases by area-level socioeconomic status among Australian-born women, and by religious affiliation across the combined sample. After adjustment for sociodemographic confounders, overseas-born women had slightly lower odds of FSD compared with Australian-born women (aOR = 0.89, 95% CI: 0.62–1.28, *p* = 0.525), however the confidence interval included the null.

**Conclusions:**

FSD affected 46.6% of survey participants overall. Future longitudinal research is needed to build a more complete understanding of sexual health equity across diverse populations.

## Introduction

Female sexual dysfunction (FSD), broadly defined as persistent difficulties with desire, arousal, orgasm, or sexual pain that cause personal distress, represents one of the most prevalent yet under-recognised conditions affecting women's quality of life and well-being (1). FSD arises from a combination of biological, psychological, and social factors, with established predictors including physical and mental health, relationship dynamics, and broader sociocultural influences (2, 3).

Global prevalence estimates range from 25.8% to 91%, a variability that reflects differences in diagnostic criteria, measurement instruments, and methods of data collection across studies (4). When attention is narrowed to studies using the same instrument, estimates converge. Two systematic reviews and meta-analyses based predominantly on the Female Sexual Function Index (FSFI) report pooled prevalence figures of 40.9% in premenopausal women (95% CI: 37.1–44.7; 95 studies) (5) and 50.75% in reproductive-aged women (95% CI: 41.73–59.78; 21 studies) (6). Both estimates are consistent with the International Consultation on Sexual Medicine consensus that 40%–50% of women report sexual complaints across the lifespan (7, 8).

In Australia, population-level data confirm that sexual difficulties are common. The Second Australian Study of Health and Relationships (ASHR2), a nationally representative survey of 20,091 adults, found that 68% of women reported at least one sexual difficulty lasting at least one month in the previous year, with lack of interest in sex (52%), inability to reach orgasm (25%), and vaginal dryness (22%) being the most common concerns (9). The Australian Women's Midlife Years (AMY) study, the largest community-based study of sexual function in midlife Australian women to date, found that nearly one in four of 5,468 women aged 40–69 years met criteria for estimated FSD, while approximately half experienced sexually related personal distress (10). Importantly, women of Asian ancestry were significantly less likely to experience arousal dysfunction and sexual self-image difficulties than women of European ancestry (10). This suggests that cultural background shapes sexual function even within the Australian context, and that ethnicity cannot be treated as a peripheral variable in sexual health research.

Despite this evidence, most studies have not been designed to capture the experiences of specific population subgroups, particularly the growing number of migrant and refugee women in Australia (11, 12). This is a significant oversight. Migrant and refugee women may face additional and intersecting vulnerabilities that heighten their risk of sexual dysfunction, including financial and language barriers, disruptions to social networks, changes in relationship dynamics, exposure to discrimination, acculturation stress, and barriers to accessing culturally safe healthcare (13). Yet studies examining these experiences remain rare, typically limited to small, single-community samples, and largely absent from the Australian context (14).

Taken together, these gaps mean that little is known about how sexual function varies across migrant groups or how migration-related factors shape sexual health outcomes in Australia. This study directly addresses that gap by comparing rates of FSD between foreign-born and Australian-born women, aiming to generate evidence that reflects the diversity of Australia's population.

## Methods

### Study design and participants

This cross-sectional study aimed to describe and compare the patterns of FSD between foreign-born and Australian-born women residing in Australia. Participants were recruited through Qualtrics, an online survey platform, using a convenience sampling approach with equal recruitment targets set for foreign-born and Australian-born women to enable balanced comparative analyses (15). Qualtrics identified eligible panellists from their profiled research panels and sent email invitations describing the study's aims. Participants who accessed the secure web-based survey via a unique URL were presented with a consent form before survey commencement; only those who provided consent proceeded to the screening questions and survey. Data collection occurred between 7 February and 7 March 2024. The dataset was verified against the predefined screening criteria and underwent standard internal quality control procedures, including automated and manual checks for bot-like behaviour, duplicate responses, and routing consistency.

Eligible participants identified as cisgender heterosexual women, were aged 18–49 years, were sexually active in the four weeks prior to survey completion and had sufficient English proficiency to complete the questionnaire. Participants were additionally excluded if they reported current pregnancy, breastfeeding, or use of medications likely to affect sexual function, including Selective Serotonin Reuptake Inhibitors (SSRIs), Serotonin and Norepinephrine Reuptake Inhibitors (SNRIs), antidepressants, antipsychotics, mood stabilisers, anti-oestrogens, GnRH agonists, hormonal treatments for PCOS or fertility, thyroxine, or a current diagnosis of cancer (16).

Aboriginal and Torres Strait Islander women were excluded from the Australian-born group. Indigenous and non-Indigenous Australian-born women represent analytically distinct populations whose health outcomes reflect fundamentally different historical and socio-political contexts, including the ongoing impacts of colonisation, dispossession, and structural racism. Sexual and reproductive health research with Indigenous women requires approaches designed by and with Indigenous communities, using methodologies that honour Indigenous sovereignty (17, 18).

### Sample size

Sample size was calculated using the Australian Bureau of Statistics (ABS) sample size calculator (19), assuming a 95% confidence level, a 5% margin of error, and an estimated FSD prevalence of 40%.This estimate was anchored on the FSFI-based meta-analysis of premenopausal women by McCool et al, which reported a pooled prevalence of 40.9% (95% CI: 37.1–44.7) and most closely matched the age range and measurement instrument used in the present study (5). It is also consistent with more recent FSFI-based meta-analyses in reproductive-aged women, which reported pooled prevalence estimates of 50.75% (6) and 47.81% (20). The 40% assumption therefore represents a conservative anchor within the FSFI-relevant literature.

The calculation yielded a required sample of 369 women per group, which was increased to 400 per group (an 8% increase) to allow for incomplete responses.

### Survey instruments

FSD was assessed using the FSFI, a validated 19-item instrument measuring sexual function over the previous four weeks across six domains: Desire, Arousal, Lubrication, Orgasm, Satisfaction, and Pain. To ensure alignment with this recall period and to avoid artificially deflated scores, an eligibility requirement for recent sexual activity was applied, preventing erroneous zero values from biasing results toward dysfunction (21). FSFI total scores range from 2.0 to 36.0, with higher scores indicating better sexual function (22, 23). The FSFI is the most widely used and validated instrument for assessing women's sexual function in clinical and population-based research (22), although two limitations have been raised in the literature. First, the original scoring procedure assigns a value of zero to women who report no sexual activity in the recall period, which biases prevalence estimates upward by classifying sexually inactive women as having dysfunction (24). To address this concern, the present study applied an eligibility requirement for recent sexual activity in the four weeks prior to survey completion, in line with current FSFI scoring guidance (21). Second, the FSFI's desire domain is anchored in a spontaneous model of sexual motivation and may not fully capture the responsive and context-dependent patterns of desire that many women describe (25). These instrument-related considerations were taken into account when interpreting the findings.

A demographic questionnaire collected information on age, education, occupation, income, residential postcode, ethnicity, primary language, religion, country of birth, parity, current pregnancy and breastfeeding status, visa and residency type, relationship status and duration, medical conditions, and medications taken. Area-level socioeconomic disadvantage was measured using the Index of Relative Socio-economic Advantage and Disadvantage (IRSAD), categorised into quintiles (26). IRSAD is developed by ABS using a wide range of area level measures such as education, income, employment, and housing etc. Region of origin was classified according to the World Health Organisation regions (27).

Country of birth was used to classify participants into two groups for comparative analysis: foreign-born women, defined as those born outside Australia and currently residing in Australia, and Australian-born women, defined as those born in Australia and currently residing in Australia.

### Statistical analyses

All statistical analyses were performed using Stata version 17 (28). Continuous variables were reported as means and standard deviations with 95% Confidence Intervals (CI), and categorical variables as frequencies and percentages. Participants who selected “Prefer not to say” were treated as missing and excluded from the respective variable-level analyses.

Group differences in FSFI domain and total scores were examined using independent samples t-tests. Chi-square tests were used to compare categorical sociodemographic characteristics between groups. Consistent with current guidance from the American Statistical Association, results are not interpreted on the basis of *p* values alone; effect sizes and confidence intervals are reported to support interpretation (29).

FSD was defined as an FSFI total score of 26.55 or below (22). Prevalence was calculated separately for foreign-born and Australian-born women and reported with 95% CI. Subgroup analyses stratified FSD prevalence by sociodemographic characteristics including age, education, employment status, income, area-level disadvantage, religion, ethnicity, and relationship status and duration. Variables specific to the migration context, including WHO region of origin, and visa status, were examined in the foreign-born group only.

To assess the robustness of findings, a pre-specified sensitivity analysis restricted the sample to responses completed between five and 40 min, excluding likely inattentive or invalid responses. FSFI scores and FSD prevalence were recalculated for this restricted sample and compared with primary estimates.

Multivariable logistic regression was performed to examine whether the similar prevalence of FSD between groups persisted after adjustment for sociodemographic differences observed at baseline. FSD was a binary variable (FSFI total score ≤ 26.55, FSD >26.55) and exposure was defined based on birthplace (foreign-born vs. Australian-born). Two models were used; an unadjusted model to estimate crude association between birthplace and FSD, and a fully adjusted model incorporating confounders selected *a priori* on the basis of established literature. Variables were included if prior evidence indicated an independent association with sexual function, migration background, or both (30). On this basis, the adjusted model included age group, education, marital status, religion, area-level socioeconomic position (IRSAD), annual household income, employment status, and gravidity (30). A supplementary model was additionally restricted to foreign-born women to examine whether visa status and region of origin independently affected the odds of FSD after accounting for sociodemographic differences. These variables were not included in the primary adjusted model as they apply exclusively to foreign-born women. Results are reported as odds ratios (OR) and adjusted odds ratios (aOR) with 95% confidence intervals.

### Ethics approval

This study was approved by the University of Adelaide Human Research Ethics Committee prior to data collection (protocol no. H-2023-264, approved 26 October 2023). Informed consent was obtained from all participants electronically. The opening page of the survey presented a consent form outlining the study's purpose, procedures, potential risks, and confidentiality assurances. Participants were required to actively confirm consent before proceeding; those who declined were automatically redirected out of the survey.

## Result

**Figure 1:**
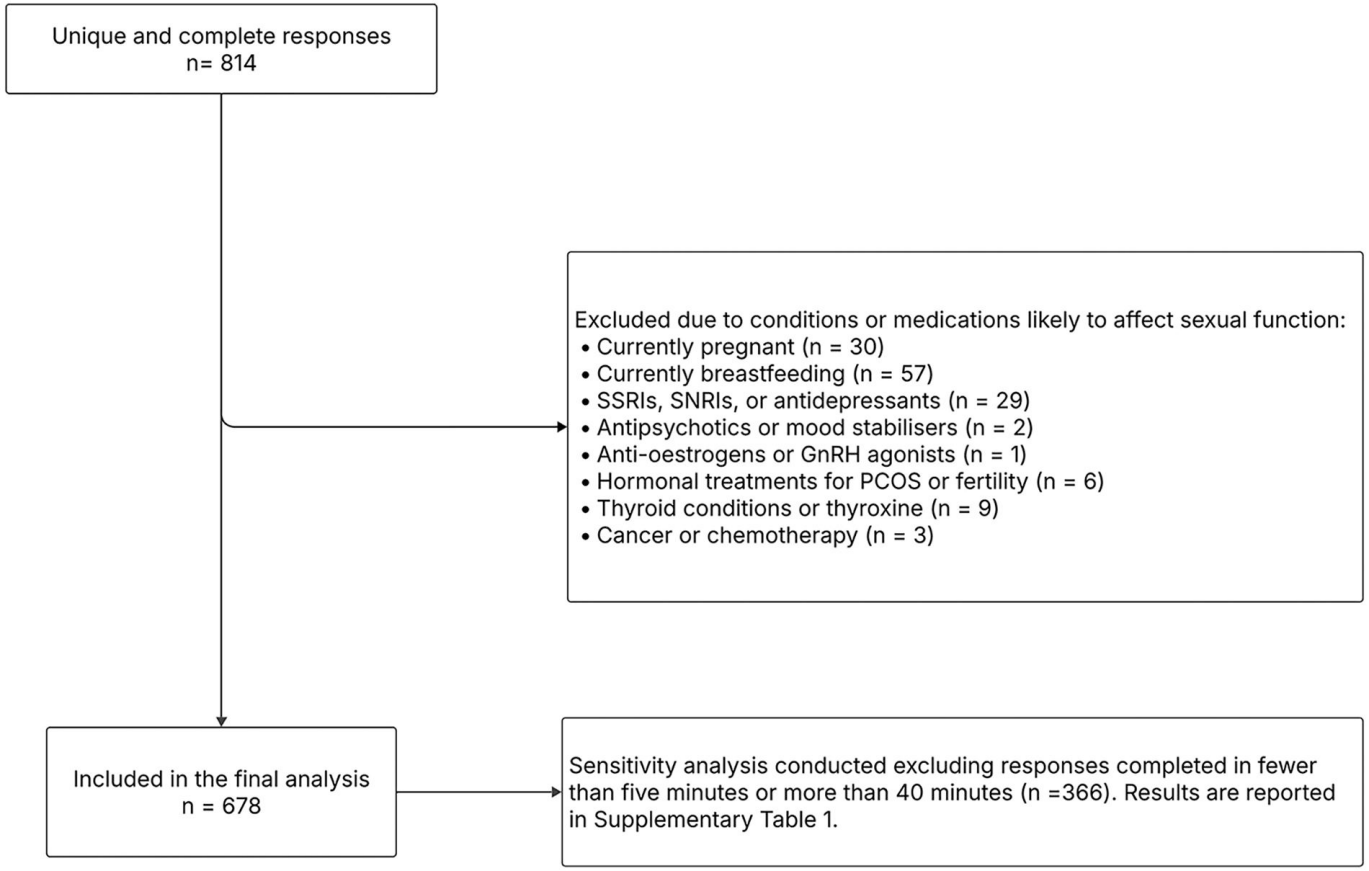
Study profile.

A total of 814 complete responses were obtained during the recruitment period. Following the application of pre-specified exclusion criteria for current pregnancy, breastfeeding, use of medications likely to affect sexual function, and a current diagnosis of cancer, 136 responses were excluded, yielding a final analytical sample of 678 women (342 Australian-born and 336 foreign-born). The participant flow is summarised in Figure 1. Participants were classified into two groups based on country of birth: Australian-born women (*n* = 342) and foreign-born women currently residing in Australia (*n* = 336). Foreign-born women originated from diverse regions, with the largest proportions from the Western Pacific (36.9%), Europe (22.92%), and South-East Asia (18.75%), and the majority held permanent residency or citizenship (76.2%). While the two groups were comparable in employment status and income, foreign-born women were more highly educated, more likely to have a partner, and resided in more socioeconomically advantaged areas. Although foreign-born women were on average 1.23 years older than Australian-born women (34.35 vs. 33.12 years; mean difference 1.23 years, 95% CI: 0.02–2.43), this difference was small in absolute terms and is unlikely to be clinically meaningful. Religious affiliation also differed markedly, with Australian-born women more commonly reporting no religious affiliation. Full sociodemographic characteristics are presented in Table 1.

**Table 1:** Demographic characteristics among Australian and foreign-born women.

Variable	Australian born (*n* = 342)	Foreign-born (*n* = 336)
Age (years), mean ± SD	33.12 ± 7.8	34.35 ± 8.12
Age category, *n* (%)		
18–29	119 (34.8)	105 (31.25)
30–39	153 (44.74)	134 (39.88)
40–49	70 (20.47)	97 (28.87)
Religion, *n* (%)		
No Religion	173 (50.58)	92 (27.38)
Catholic	56 (16.37)	69 (20.54)
Christian	56 (16.37)	77 (22.92)
Atheist	36 (10.53)	17 (5.06)
Hinduism	3 (0.88)	26 (7.74)
Islam	4 (1.17)	23 (6.85)
Buddhism	2 (0.58)	18 (5.36)
Other	12 (3.51)	14 (4.17)
Marital status, *n* (%)	*n* = 340	*n* = 333
Partnered	230 (67.25)	256 (76.19)
Single (never married)	99 (28.95)	66 (19.64)
Previously partnered	11 (3.22)	11 (3.27)
Education, *n* (%)		
Secondary or less	216 (53.07)	102 (25.44)
Bachelor's degree	143 (35.14)	208 (51.87)
Postgraduate	48 (11.79)	91 (22.69)
Employment status, *n* (%)		
Employed	272 (79.53)	264 (78.57)
Student	9 (2.63)	17 (5.06)
Unemployed	61 (17.84)	55 (16.37)
Annual income, *n* (%)	*n* = 323	*n* = 318
Low (<$40,000)	46 (14.24)	26 (8.18)
Lower–middle ($40,000–$79,999)	84 (26.10)	87 (27.36)
Upper–middle ($80,000–$149,999)	121 (37.46)	130 (40.88)
High ($150,000+)	72 (22.29)	75 (23.58)
Area-level socioeconomic status (IRSAD), *n* (%)	*n* = 339	*n* = 335
Most disadvantaged	65 (19.01)	30 (8.93)
Disadvantaged	55 (16.08)	31 (9.23)
Middle	61 (17.84)	61 (18.15)
Advantaged	76 (22.22)	96 (28.57)
Most advantaged	82 (23.98)	117 (34.82)
Number of pregnancies, *n* (%)		
Nulligravida (0)	132 (38.6)	145 (43.15)
One pregnancy	67 (19.59)	66 (19.64)
Two pregnancies	62 (18.13)	79 (23.51)
Three or more	81 (23.68)	46 (13.69)
Ethnicity, *n* (%)		
Caucasian	–	100 (29.76)
Southeast Asian	–	89 (26.49)
South Asian	–	46 (13.69)
East Asian	–	32 (9.52)
Middle Eastern	–	23 (6.85)
African	–	16 (4.76)
Pacific Islander	–	16 (4.76)
Latin American	–	13 (3.87)
Other (e.g., mixed)	–	1 (0.3)
WHO Region, *n* (%)		
Western Pacific Region	–	124 (36.9)
European Region	–	77 (22.92)
South-East Asia Region	–	63 (18.75)
Eastern Mediterranean Region	–	27 (8.04)
African Region	–	22 (6.55)
Region of the Americas	–	20 (5.95)
Other	–	3 (0.89)
Visa status, *n* (%)		
Permanent residency	–	196 (58.33)
Citizen	–	60 (17.86)
Work visa	–	40 (11.9)
Student visa	–	30 (8.93)
Other	–	10 (2.98)

Mean ± standard deviation (SD), compared using independent samples *t*-test. Frequency (*n*, %), compared using chi-square test. IRSAD: Index of Relative Socio-economic Advantage and Disadvantage.

Note: Variables reported for foreign-born women only. Participants who selected “Prefer not to say” were treated as missing and excluded from variable-level analyses. Valid response rates for each variable are reported alongside the respective variable.

Mean FSFI domain and total scores were comparable between Australian-born and foreign-born women across all domains (Table 2). Pain scores were highest in both groups (Australian-born: 4.71 ± 1.46; foreign-born: 4.73 ± 1.36), while desire scores were lowest (Australian-born: 3.82 ± 1.25; foreign-born: 3.77 ± 1.10), consistent with patterns reported in the broader literature. The largest observed domain difference was in Satisfaction, where foreign-born women scored marginally higher than Australian-born women (4.59 ± 1.19 vs. 4.43 ± 1.18; difference = 0.16, 95% CI: −0.02 to 0.34;), although the magnitude of difference is very small.

**Table 2:** FSFI domains and total score among Australian and foreign born women.

FSFI domain mean ± SD (95% CI)	Australian-born (*n* = 342) Mean ± SD [95% CI]	Foreign-born (*n* = 336) Mean ± SD [95% CI]	*P*-value
Desire	3.82 ± 1.25 [3.69, 3.95]	3.77 ± 1.10 [3.65, 3.89]	0.583
Arousal	4.46 ± 1.31 [4.33, 4.60]	4.45 ± 1.18 [4.33, 4.58]	0.913
Lubrication	4.61 ± 1.33 [4.47, 4.76]	4.64 ± 1.29 [4.50, 4.78]	0.802
Orgasm	4.31 ± 1.47 [4.15, 4.46]	4.23 ± 1.38 [4.08, 4.38]	0.491
Satisfaction	4.43 ± 1.18 [4.30, 4.55]	4.59 ± 1.19 [4.46, 4.72]	0.078
Pain	4.71 ± 1.46 [4.56, 4.87]	4.73 ± 1.36 [4.58, 4.88]	0.863
FSFI total score	26.35 ± 5.72 [25.74, 26.95]	26.41 ± 5.69 [25.80, 27.03]	0.876
FSD, *n* (%) [95% CI]	161 (47.1%) [41.7, 52.5]	155 (46.1%) [40.7, 51.6]	0.805

FSFI, Female Sexual Function Index; FSD, Female Sexual Dysfunction; SD, Standard Deviation; CI, Confidence Interval.

Continuous variables compared using independent samples t-test; categorical variables compared using chi-square test. *p* values reported to three decimal places.

Higher FSFI domain and total scores indicate better sexual function. FSD defined as an FSFI total score 26.55 or below.

Mean FSFI total scores were nearly identical between groups (Australian-born: 26.35 ± 5.72; foreign-born: 26.41 ± 5.69; difference = 0.06, 95% CI: −0.70 to 0.82). However, both means fall within less than half a point of the 26.55 threshold, which means that a substantial proportion of participants scored in a narrow band around the cut-off where the distinction between dysfunction and normal function is clinically ambiguous. In practical terms, small shifts in the threshold value would reclassify a considerable number of women in either direction, and the prevalence estimates should be interpreted with this sensitivity in mind. The near-identical positioning of both group means relative to the threshold further reinforces that the two groups are indistinguishable in terms of overall sexual function. The estimate of FSD was similarly high in both groups, affecting 47.1% of Australian-born respondents (95% CI: 41.7–52.5) and 46.1% of foreign-born participants (95% CI: 40.7–51.6; difference = 1.0 percentage point).

Sociodemographic characteristics of Australian-born and foreign-born women with FSD are presented in Table 3. There were no meaningful differences between Australian-born and foreign-born survey participants with FSD in terms of age, education, employment status, income, parity, or relationship duration. However, there were differences in terms of socioeconomic disadvantage and religious affiliation. Among Australian-born women with FSD, the distribution across socioeconomic areas was broad. The largest proportions resided in advantaged (26.1%, 95% CI: 19.5–33.6%) and most advantaged (26.1%, 95% CI: 19.5–33.6%) areas, while 22.4% (95% CI: 16.2–29.6%) resided in the most disadvantaged areas. Among foreign-born women with FSD, the distribution was more concentrated in advantaged (30.3%, 95% CI: 23.2–38.2%) and most advantaged (36.8%, 95% CI: 29.2–44.9%) areas, with a smaller proportion residing in the most disadvantaged areas (7.7%, 95% CI: 4.1–13.1%).

**Table 3:** Sociodemographic characteristics of the Australian-born and foreign-born women with FSD.

Variable	Overall FSD (*n* = 316) *n* (%) [95% CI]	Australia-born FSD (*n* = 161) *n* (%) [95% CI]	Foreign-born FSD (*n* = 155) *n* (%) [95% CI]
Age group			
18–29	106 (33.5%) [28.4–39.0]	60 (37.3%) [29.8–45.2]	46 (29.7%) [22.6–37.5]
30–39	130 (41.1%) [35.7–46.8]	69 (42.9%) [35.1–50.9]	61 (39.4%) [31.6–47.5]
40–49	80 (25.3%) [20.6–30.5]	32 (19.9%) [14.0–26.9]	48 (31.0%) [23.8–38.9]
Education			
Secondary or less	125 (39.6%) [34.1–45.2]	80 (49.7%) [41.7–57.7]	45 (29.0%) [22.0–36.9]
Bachelor's degree	143 (45.3%) [39.7–50.9]	60 (37.3%) [29.8–45.2]	83 (53.5%) [45.4–61.6]
Postgraduate	48 (15.2%) [11.4–19.6]	21 (13.0%) [8.3–19.2]	27 (17.4%) [11.8–24.3]
Employment status			
Employed	252 (79.7%) [74.9–84.0]	130 (80.7%) [73.8–86.5]	122 (78.7%) [71.4–84.9]
Student	14 (4.4%) [2.4–7.3]	5 (3.1%) [1.0–7.1]	9 (5.8%) [2.7–10.7]
Unemployed	50 (15.8%) [12.0–20.3]	26 (16.1%) [10.8–22.8]	24 (15.5%) [10.2–22.2]
Annual Income	*n* = 298	*n* = 152	*n* = 146
Low (<$40,000)	42 (13.3%) [9.7–17.5]	26 (16.1%) [10.8–22.8]	16 (10.3%) [6.0–16.2]
Lower–middle ($40,000–$79,999)	75 (23.7%) [19.2–28.8]	35 (21.7%) [15.6–28.9]	40 (25.8%) [19.1–33.4]
Upper–middle ($80,000–$149,999)	114 (36.1%) [30.8–41.6]	56 (34.8%) [27.5–42.7]	58 (37.4%) [29.8–45.5]
High ($150,000+)	67 (21.2%) [16.8–26.1]	35 (21.7%) [15.6–28.9]	32 (20.6%) [14.6–27.9]
Area-level socioeconomic status (IRSAD)	*n* = 315	*n* = 160	*n* = 155
Most disadvantaged	48 (15.2%) [11.4–19.6]	36 (22.4%) [16.2–29.6]	12 (7.7%) [4.1–13.1]
Disadvantaged	29 (9.2%) [6.2–12.9]	19 (11.8%) [7.3–17.8]	10 (6.5%) [3.1–11.5]
Middle	50 (15.8%) [12.0–20.3]	21 (13.0%) [8.3–19.2]	29 (18.7%) [12.9–25.8]
Advantaged	89 (28.2%) [23.3–33.5]	42 (26.1%) [19.5–33.6]	47 (30.3%) [23.2–38.2]
Most advantaged	99 (31.3%) [26.3–36.8]	42 (26.1%) [19.5–33.6]	57 (36.8%) [29.2–44.9]
Marital status	*n* = 313	*n* = 160	*n* = 153
Partnered	225 (71.2%) [65.9–76.1]	106 (65.8%) [58.0–73.1]	119 (76.8%) [69.3–83.2]
Single (never married)	83 (26.3%) [21.5–31.5]	51 (31.7%) [24.6–39.5]	32 (20.6%) [14.6–27.9]
Previously partnered	5 (1.6%) [0.5–3.7]	3 (1.9%) [0.4–5.3]	2 (1.3%) [0.2–4.6]
Relationship duration (partnered only)	*n* = 225	*n* = 106	*n* = 119
<2 years	3 (0.9%) [0.2–2.7]	0 (0.0%) [0.0–2.3]	3 (1.9%) [0.4–5.6]
2–4 years	38 (12.0%) [8.7–16.1]	21 (13.0%) [8.3–19.2]	17 (11.0%) [6.5–17.0]
5–9 years	69 (21.8%) [17.4–26.8]	32 (19.9%) [14.0–26.9]	37 (23.9%) [17.4–31.4]
10 + years	115 (36.4%) [31.1–42.0]	53 (32.9%) [25.7–40.8]	62 (40.0%) [32.2–48.2]
Number of pregnancies			
Nulligravida (0)	125 (39.6%) [34.1–45.2]	61 (37.9%) [30.4–45.9]	64 (41.3%) [33.5–49.5]
One pregnancy	68 (21.5%) [17.1–26.5]	37 (23.0%) [16.7–30.3]	31 (20.0%) [14.0–27.2]
Two pregnancies	65 (20.6%) [16.2–25.5]	28 (17.4%) [11.9–24.1]	37 (23.9%) [17.4–31.4]
Three or more	58 (18.4%) [14.2–23.1]	35 (21.7%) [15.6–28.9]	23 (14.8%) [9.6–21.4]
Religion			
No Religion	126 (39.9%) [34.4–45.5]	86 (53.4%) [45.4–61.3]	40 (25.8%) [19.1–33.4]
Christian	66 (20.9%) [16.5–25.8]	28 (17.4%) [11.9–24.1]	38 (24.5%) [18.0–32.1]
Catholic	46 (14.6%) [10.9–18.9]	22 (13.7%) [8.8–20.0]	24 (15.5%) [10.2–22.2]
Atheist	28 (8.9%) [6.0–12.6]	18 (11.2%) [6.8–17.1]	10 (6.5%) [3.1–11.5]
Hinduism	16 (5.1%) [2.9–8.1]	2 (1.2%) [0.2–4.4]	14 (9.0%) [5.0–14.7]
Buddhism	14 (4.4%) [2.4–7.3]	0 (0.0%) [0.0–2.3]	14 (9.0%) [5.0–14.7]
Islam	12 (3.8%) [2.0–6.5]	2 (1.2%) [0.2–4.4]	10 (6.5%) [3.1–11.5]
Other	8 (2.5%) [1.1–4.9]	3 (1.9%) [0.4–5.3]	5 (3.2%) [1.1–7.4]
WHO region (foreign-born only)			
Western Pacific Region			56 (36.1%) [28.6–44.2]
South-East Asia Region			35 (22.6%) [16.3–30.0]
European Region			30 (19.4%) [13.5–26.5]
Eastern Mediterranean Region			13 (8.4%) [4.5–13.9]
African Region			12 (7.7%) [4.1–13.1]
Region of the Americas			9 (5.8%) [ 2.7–10.7]
Ethnicity (foreign-born only)			
Southeast Asian			42 (27.1%) [20.3–34.8]
Caucasian			43 (27.7%) [20.9–35.5]
South Asian			25 (16.1%) [10.7–22.9]
East Asian			18 (11.6%) [7.0–17.7]
Middle Eastern			12 (7.7%) [4.1–13.1]
African			6 (3.9%) [1.4–8.2]
Latin American			5 (3.2%) [1.1–7.4]
Pacific Islander			4 (2.6%) [0.7–6.5]
Visa status (foreign-born only)			
Permanent residency			99 (63.9%) [55.8–71.4]
Citizen			24 (15.5%) [10.2–22.2]
Student visa			16 (10.3%) [6.0–16.2]
Work visa			15 (9.7%) [5.5–15.5]
Other			1 (0.6%) [0.0–3.5]

Column percentages are calculated among women with FSD within each group. Relationship duration is restricted to partnered women only. WHO region, ethnicity, and visa status are reported for foreign-born women only. FSD defined as FSFI total score 26.55 or below. 95% CI calculated using the exact binomial method.

Among Australian-born women with FSD, the majority reported no religious affiliation (53.4%, 95% CI: 45.4–61.3%), while among foreign-born women with FSD, the distribution was more varied, with no religion (25.8%, 95% CI: 19.1–33.4%), Christianity (24.5%, 95% CI: 18.0–32.1%), and Catholicism (15.5%, 95% CI: 10.2–22.2%) being the most common affiliations.

Among foreign-born women with FSD the largest proportion were permanent residents (63.9%, 95% CI: 55.8–71.4). FSD distribution did not differ meaningfully by WHO region of origin (*p* = 0.308) or ethnicity (*p* = 0.447).

FSFI total scores and FSD prevalence remained comparable between groups in the sensitivity-restricted sample (responses completed between five and 40 min, *n* = 312; Supplementary Table S1). FSFI domain and total scores remained comparable between Australian-born and foreign-born women across most domains. The satisfaction domain showed a difference of 0.29 points in favour of foreign-born women (4.78, 95% CI: 4.61–4.95 vs. 4.49, 95% CI: 4.28–4.71), consistent with the borderline pattern observed in the primary analysis. Mean FSFI total scores were similar between groups (Australian-born: 26.84 ± 5.61; foreign-born: 27.17 ± 5.59; difference = 0.33), and FSD prevalence remained comparable (Australian-born: 37.4%, 95% CI: 29.4–46.2; foreign-born: 39.7%, 95% CI: 33.0–46.8). These findings support the robustness of the primary results.

In the unadjusted model, overseas-born women did not differ from Australian-born women in their odds of FSD (OR=0.96, 95% CI: 0.71–1.30, *p* = 0.805). After adjustment for age, education, marital status, religion, IRSAD, gravidity, annual household income, and employment status, the pattern remained almost similar (aOR = 0.88, 95% CI: 0.62–1.28, *p* = 0.525) with slightly lower odds of FSD among overseas born compared with Australian-born women, however, the confidence interval included the null.

Among the covariates in the adjusted model, two showed independent associations with FSD. Previously partnered women had lower odds of FSD than partnered women (aOR = 0.32, 95% CI: 0.11–0.92, *p* = 0.035), women who had experienced one pregnancy had higher odds of FSD than nulligravid women (aOR = 1.68, 95% CI: 1.04–2.71, *p* = 0.033). Religious affiliation, area-level socioeconomic position, age group, education, annual household income, and employment status were not independently associated with FSD in the adjusted model. Full results are presented in Table 4.

**Table 4:** Adjusted odds ratios for female sexual dysfunction among Australian-born and foreign-born women (*n* = 634).

Variable	Adjusted OR [95% CI]
Birthplace	
Australian-born women	Reference
Overseas-born women	0.89 [0.62–1.28]
Age group	
18–29 years	Reference
30–39 years	0.97 [0.64–1.47]
40–49 years	1.16 [0.70–1.92]
Education	
Secondary or less	Reference
Bachelor's degree	0.93 [0.62–1.38]
Postgraduate	0.82 [0.49–1.38]
Employment status	
Employed	Reference
Student	1.34 [0.51–3.49]
Unemployed	0.81 [0.50–1.32]
Religion	
No Religion	Reference
Atheist	1.34 [0.70–2.54]
Buddhism	2.62 [0.85–8.03]
Catholic	0.70 [0.43–1.14]
Christian	1.16 [0.73–1.84]
Hinduism	1.69 [0.73–3.88]
Islam	1.00 [0.42–2.38]
Other	0.48 [0.20–1.18]
IRSAD	
Most disadvantaged	Reference
Disadvantaged	0.60 [0.32–1.15]
Middle	0.76 [0.42–1.38]
Advantaged	1.46 [0.83–2.56]
Most advantaged	1.15 [0.65–2.02]
Annual income	
Low (<$40,000)	Reference
Lower-middle ($40,000–$79,999)	0.65 [0.36–1.18]
Upper-middle ($80,000–$149,999)	0.62 [0.34–1.13]
High ($150,000+)	0.60 [0.31–1.19]
Marital status	
Partnered	Reference
Single (never married)	1.23 [0.79–1.92]
Previously partnered	0.32 [0.11–0.92]
Gravidity	
Nulligravida	Reference
One pregnancy	1.68 [1.04–2.71]
Two pregnancies	1.34 [0.81–2.21]
Three or more	1.34 [0.79–2.26]

OR, Odds Ratio; CI, Confidence Interval; IRSAD, Index of Relative Socio-economic Advantage and Disadvantage; FSD defined as FSFI total score ≤26.55. Confounders were selected *a priori* based on established literature and subject matter knowledge. The adjusted model controls for age, education, marital status, religion, IRSAD, gravidity, annual household income, and employment status. Ethnicity, WHO region of origin, and visa status were not included as they apply exclusively to foriegn-born women and are examined in the supplementary model (Supplementary Table S2).

Note: Buddhism estimate based on *n* = 20 women; interpret with caution due to small cell size and wide confidence interval.

A supplementary adjusted model was restricted to overseas-born women (*n* = 311), additionally adjusting for visa status and WHO region of origin alongside the covariates included in the primary model. Compared with citizens, the adjusted odds of FSD did not differ significantly for women holding permanent residency (aOR = 1.32, 95% CI: 0.67–2.60, *p* = 0.427), those on a student visa (aOR = 1.05, 95% CI: 0.30–3.69, *p* = 0.942), or a work visa (aOR = 0.65, 95% CI: 0.23–1.81, *p* = 0.411). WHO region of origin was likewise not independently associated with FSD in any category. Full results are presented in Supplementary Table S2.

## Discussion

This study found that almost half of the survey respondents had FSD with estimates of 47.1% among Australian-born women and 46.1% among foreign-born women having FSD, with no meaningful difference between the groups. Mean FSFI total scores were nearly identical and fell below the validated dysfunction threshold in both groups, indicating that the average woman in each group met the criterion for FSD regardless of birthplace. Across all six FSFI domains scores were comparable, except for a borderline difference in sexual Satisfaction favouring foreign-born women.

The estimates observed in both groups is consistent with published estimates for reproductive-aged women. The most recent systematic review and meta-analysis which pooled data from 36,777 women across 20 studies published between 2015 and 2024, estimated a pooled FSD prevalence of 47.81% (95% CI: 39.19–56.43%) (20), a figure that closely mirrors both the Australian-born and foreign-born prevalence observed in the present study. Another meta-analysis of 12,504 women reported a pooled prevalence of 50.75% (95% CI: 41.73–59.78) (6). These findings also fall within the 40%–50% range established by the Fourth International Consultation on Sexual Medicine (7). That both group means cluster just below the threshold also highlights the well-recognised sensitivity of the 26.55 cut-off, and suggests that the high prevalence observed in this study partly reflects the positioning of the diagnostic boundary within a densely populated region of the score distribution.

Despite this alignment with broader prevalence estimates, the similar prevalence of FSD between groups challenges assumptions that migrant women are inherently more vulnerable to sexual dysfunction than their native-born counterparts. A scoping review of sexual function in migrant and refugee women found that migrant women consistently reported lower levels of sexual Desire and Arousal compared with non-migrant women, with acculturation stress, conservative sexual beliefs, and post-migration difficulties identified as key contributing factors (31). However, several characteristics of the present sample may help explain why these anticipated differences did not emerge.

A first consideration relates to who were reached by the study. The English language requirement and recruitment through an online panel are likely to have narrowed the foreign-born sample to women who were already digitally connected and well-integrated into life in Australia. This is reflected in the sample profile, in which the majority of foreign-born women held permanent residency or citizenship, were highly educated, and resided in socioeconomically advantaged areas. A foreign-born sample shaped in this way is unlikely to capture the experiences of women whose circumstances would be expected, on the basis of the broader migrant health literature, to confer greater sexual health risk. The convergence in FSD prevalence between groups may therefore reflect, in part, the sociodemographic similarity and online recruitment of educated, digitally literate and socioeconomically advantaged foreign-born women.

A further consideration concerns whether the sociodemographic differences between groups could themselves explain the similar prevalence of FSD. Foreign-born women in this sample were more highly educated, more likely to be partnered, and more concentrated in advantaged area-level socioeconomic quintiles, all of which are independently associated with better sexual function in the literature (32–35). After adjustment for age, education, marital status, religion, area-level socioeconomic position, gravidity, annual household income, and employment status in a multivariable logistic regression model, the odds of FSD among foreign-born women did not significantly differ from those of Australian-born women (aOR = 0.89, 95% CI: 0.62–1.28, *p* = 0.525).

Set against the broader literature on migrant health, this pattern is also consistent with the healthy immigrant phenomenon, in which positively selected migrants exhibit better health outcomes than domestic-born populations in the destination country (36), although the present cross-sectional design and the absence of measures of general health and pre-migration characteristics mean this interpretation must remain tentative.

Further explanation relates to the immigration status of foreign-born participants in this sample. Evidence from natural experiments shows that restrictive migration policies and insecure visa status are associated with poorer health outcomes among migrants, whereas secure immigration status can have a protective effect on health and wellbeing (37). In this study, the majority of foreign-born women held permanent residency or citizenship, indicating a relatively stable and secure position with respect to their immigration status. This stability is relevant in two ways. First, it suggests that the foreign-born group in this sample was largely buffered from the structural and psychosocial stressors, including financial precarity, fear of deportation, and restricted healthcare access, that are typically associated with poorer sexual health outcomes in migrant populations (38). Second, it is consistent with the attenuation of the healthy immigrant effect described above, as permanently settled women are likely to have resided in Australia for extended periods, during which any initial health advantage may have diminished. Together, these factors help explain why FSD prevalence converged between groups rather than diverging as the broader migrant health literature might predict.

The borderline higher Satisfaction score observed among foreign-born women in both the primary and sensitivity analyses is worth interpreting carefully. Unlike the other FSFI domains, which capture more physiologically anchored aspects of sexual function, Satisfaction is a subjective appraisal of one's sexual relationship and is therefore shaped by expectations, relational dynamics, and cultural reference points (21, 39). Foreign-born women in this sample were more likely to be partnered than Australian-born women (76.2% vs. 67.3%), and partnership has been consistently linked with higher sexual satisfaction in the literature (32–35).

It is also plausible that women from cultural backgrounds in which sexual experience is framed within different relational, normative, or religious contexts evaluate their experiences against different benchmarks, which may translate into higher reported satisfaction even where scores across the other domains are comparable (35, 40). Given the borderline statistical signal and the absence of direct measures of relationship quality or cultural sexual norms in this study, this interpretation remains tentative, and dedicated qualitative and mixed-methods research will be needed to examine these mechanisms more directly.

Patterns of FSD distribution by religious affiliation differed in unadjusted comparisons across the combined sample, although no individual religious category was independently associated with FSD after adjustment. The relationship between religion and sexual function is well recognised as complex and context-dependent, with higher religious commitment associated with both more conservative sexual attitudes and, in some contexts, greater relational satisfaction, with effects varying substantially by tradition and cultural background (41, 42).

Taken together, these findings suggest that sexual dysfunction is a universal burden among reproductive-aged women in Australia, cutting across birthplace, migration background, and socioeconomic position. Migration background alone is not a reliable indicator of sexual health risk, and clinicians should adopt universal screening approaches rather than using birthplace as a proxy for vulnerability. At a systems level, these findings support the integration of sexual health assessment into routine primary and reproductive healthcare, with culturally responsive services available to those who require them.

Despite these contributions, several limitations should be considered. Regarding generalisability, recruitment through an online panel with an English language requirement systematically excluded recently arrived migrants, refugees, and women with limited English proficiency, who may carry the greatest sexual health burden; findings therefore apply only to more acculturated, English-proficient foreign-born women.

Beyond language, online panel recruitment introduces further self-selection effects that warrant explicit consideration. Panel participants tend to be more digitally literate and more familiar with survey-based research than the broader population they are intended to represent, and the sensitive nature of sexual function as a topic adds a further self-selection layer, with women who are more open to discussing their sexual experiences more likely to opt in. Women who are most vulnerable to sexual dysfunction, including those who are socially isolated, in distress, or less comfortable engaging with sexual health topics, may therefore be underrepresented in the sample, which may have contributed to an underestimation of prevalence in both groups.

The exclusion of Aboriginal and Torres Strait Islander women means findings also apply only to non-Indigenous Australian-born women; Indigenous women's sexual health experiences are shaped by distinct historical, cultural, and structural factors that warrant dedicated research conducted by and with Indigenous communities. The restriction to cisgender heterosexual women further limits applicability to gender-diverse and sexual minority populations.

Regarding study design, the cross-sectional design precludes causal inference, and the self-reported nature of the data introduces the possibility of response bias, particularly given the sensitive nature of sexual function questions. The absence of data on length of residency is a notable gap, as time since migration is a key determinant of acculturation and may meaningfully shape sexual health outcomes.

Finally, while the overall sample was adequately powered to estimate FSD prevalence, the study was not powered to detect statistically significant differences within specific subgroups; several odds ratios in the multivariable model showed notable trends that did not reach statistical significance, likely reflecting reduced statistical power within smaller subgroups rather than a true absence of association. Future research should prioritise inclusive sampling strategies, longitudinal designs, and language-accessible recruitment, should stratify foreign-born women by length of residence to examine whether FSD prevalence converges with that of the host population over time, and should employ larger samples to examine subgroup-level associations with adequate statistical power.

## Conclusion

This study found that FSD affected 46.6% of reproductive-aged women surveyed in Australia, with near-identical estimates among Australian-born (47.1%) and foreign-born (46.1%) women. These findings challenge the assumption that migrant women are inherently more vulnerable to sexual dysfunction and suggest that positive selection processes and secure immigration status may buffer against the migration-related stressors that the broader literature associates with poorer sexual health. The high burden identified across both groups underscores the need for universal, culturally responsive sexual health screening in primary and reproductive healthcare settings, regardless of a woman's birthplace. Longitudinal studies will be essential to advancing a more complete understanding of sexual health equity across Australia's diverse population.

## Data Availability

The dataset analysed in this study contains sensitive information about participants' sexual health, demographic characteristics, and residential locations. In accordance with ethics approval conditions and to protect participant privacy, the dataset is not publicly available. Reasonable requests for de-identified data may be directed to the corresponding author and will be considered following appropriate ethics approvals and data sharing agreements.
