# Supplementary material for: Female sexual dysfunction among foreign and Australian-born women: a cross-sectional study
Source: Front Reprod Health. 2026 Jun 16;8:1855626. doi: 10.3389/frph.2026.1855626 (PMC13314969; doi:10.3389/frph.2026.1855626)
Supplement: Supplementary file 1 [file Table1.docx]

**Supplementary Material 1: Survey Instrument**

**Preamble Questionnaire**

The survey begins with a few simple demographic questions about you.

SCREENING QUESTIONS

1. which age group do you belong?
2. Under 18 years (ineligible - screen out)
3. 18 – 49 years
4. Over 49 years (ineligible - screen out)
5. How do you identify yourself?
6. Female
7. Male (ineligible - screen out)
8. Other (ineligible - screen out)
9. Prefer not to say (ineligible - screen out)
10. How do you identify your sexual orientation?
11. Heterosexual
12. Homosexual (ineligible - screen out)
13. Bisexual (ineligible - screen out)
14. Other (ineligible - screen out)
15. Prefer not to say (ineligible - screen out)
16. Which of the following best describes you?

a) I have migrated to Australia.

b) I was born in Australia; my parents migrated to Australia (ineligible - screen out).

c) I identify myself as Australian, I were born and raised in Australia.

d) I am of Aboriginal and Torres Strait Islander heritage (ineligible - screen out).

1. Which of the following best describes you?
2. Not sexually active (ineligible - screen out)
3. Sexually active in past 4 weeks
4. Are you currently living in Australia?
5. No (ineligible - screen out)
6. Yes

**Eligible participants will proceed to the survey:**

**Preamble Questionnaire**

The survey begins with a few simple demographic questions about you.

SCREENING QUESTIONS

1. which age group do you belong?
2. Under 18 years (ineligible - screen out)
3. 18 – 49 years
4. Over 49 years (ineligible - screen out)
5. How do you identify yourself?
6. Female
7. Male (ineligible - screen out)
8. Other (ineligible - screen out)
9. Prefer not to say (ineligible - screen out)
10. How do you identify your sexual orientation?
11. Heterosexual
12. Homosexual (ineligible - screen out)
13. Bisexual (ineligible - screen out)
14. Other (ineligible - screen out)
15. Prefer not to say (ineligible - screen out)
16. Which of the following best describes you?

a) I have migrated to Australia.

b) I was born in Australia; my parents migrated to Australia (ineligible - screen out).

c) I identify myself as Australian, I were born and raised in Australia.

d) I am of Aboriginal and Torres Strait Islander heritage (ineligible - screen out).

1. Which of the following best describes you?
2. Not sexually active (ineligible - screen out)
3. Sexually active in past 4 weeks
4. Are you currently living in Australia?
5. No (ineligible - screen out)
6. Yes

**Demographic Questions**

Background information:

1. What is your age? _____________
2. What is your country of birth?_____________________
3. Please specify your ethnicity.
4. Caucasian
5. Aboriginal or Torres Strait Islander
6. South Asian
7. East Asian
8. Southeast Asian
9. Pacific Islander
10. Middle Eastern
11. African
    1. Other – specify: _________________
12. What type of visa do you have? _________
13. What is the postcode where you are living? _________
14. What language does your family primarily speak? _________
15. What is your religious affiliation/belief? _________
16. What is your marital status?
17. Single, never married
18. Married or domestic partnership
19. Widowed
20. Divorced
21. Separated
22. Prefer not to answer
23. How ling have you been in your current relationship? _________
24. Employment Status: Are you currently?
25. Employed for wages
26. Self-employed
27. Out of work and looking for work
28. Out of work but not currently looking for work
29. A homemaker
30. A student
31. Military
32. Early Retirement
33. Unable to work due to a disability/Medical condition/caring for some one
34. What is the highest level of education you completed?
    1. No formal Education
    2. Completed year 12.
    3. TAFE Certificate or Diploma
    4. bachelor’s degree
    5. master’s or PhD degree

1. How many pregnancies have you had?
   1. Zero
   2. 1-2
   3. 3 or more
2. Are you currently breastfeeding?

a) Yes

b) No

1. Are you currently pregnant?

a) Yes

b) No

1. Do you have any medical conditions?
   1. No
   2. Yes – specify: _________________
2. Are you taking any prescribed medication/s?

a. No

b. Yes – specify: ______________

**Female Sexual Function Index (FSFI)**

INSTRUCTIONS: These questions ask about your sexual feelings and responses during the past 4 weeks. Please answer the following questions as honestly and clearly as possible. Your responses will be kept completely confidential. In answering these questions the following definitions apply:

Sexual activity can include caressing, foreplay, masturbation and vaginal intercourse.

Sexual intercourse is defined as penile penetration (entry) of the vagina.

Sexual stimulation includes situations like foreplay with a partner, self-stimulation (masturbation), or sexual fantasy.

**CHECK ONLY ONE BOX PER QUESTION.**

Sexual desire or interest is a feeling that includes wanting to have a sexual experience, feeling receptive to a partner's sexual initiation, and thinking or fantasizing about having sex.

1. Over the past 4 weeks, how **often** did you feel sexual desire or interest?

Almost always or always

Most times (more than half the time)

Sometimes (about half the time)

A few times (less than half the time)

Almost never or never

1. Over the past 4 weeks, how would you rate your **level** (degree) of sexual desire or interest?

Very high

High

Moderate

Low

Very low or none at all

Sexual arousal is a feeling that includes both physical and mental aspects of sexual excitement. It may include feelings of warmth or tingling in the genitals, lubrication (wetness), or muscle contractions.

1. Over the past 4 weeks, how **often** did you feel sexually aroused ("turned on") during sexual activity or intercourse?

No sexual activity

Almost always or always

Most times (more than half the time)

Sometimes (about half the time)

A few times (less than half the time)

Almost never or never

1. Over the past 4 weeks, how would you rate your **level** of sexual arousal ("turn on") during sexual activity or intercourse?

No sexual activity

Very high

High

Moderate

Low

Very low or none at all

1. Over the past 4 weeks, how **confident** were you about becoming sexually aroused during sexual activity or intercourse?

No sexual activity

Very high confidence

High confidence Moderate confidence Low confidence

Very low or no confidence

1. Over the past 4 weeks, how **often** have you been satisfied with your arousal (excitement) during sexual activity or intercourse?

No sexual activity

Almost always or always

Most times (more than half the time)

Sometimes (about half the time)

A few times (less than half the time)

Almost never or never

1. Over the past 4 weeks, how **often** did you become lubricated ("wet") during sexual activity or intercourse?

No sexual activity

Almost always or always

Most times (more than half the time)

Sometimes (about half the time)

A few times (less than half the time)

Almost never or never

1. Over the past 4 weeks, how **difficult** was it to become lubricated ("wet") during sexual activity or intercourse?

No sexual activity

Extremely difficult or impossible

Very difficult

Difficult Slightly difficult Not difficult

1. Over the past 4 weeks, how often did you **maintain** your lubrication ("wetness") until completion of sexual activity or intercourse?

No sexual activity

Almost always or always

Most times (more than half the time)

Sometimes (about half the time)

A few times (less than half the time)

Almost never or never

1. Over the past 4 weeks, how **difficult** was it to maintain your lubrication ("wetness") until completion of sexual activity or intercourse?

No sexual activity

Extremely difficult or impossible

Very difficult

Difficult Slightly difficult Not difficult

1. Over the past 4 weeks, when you had sexual stimulation or intercourse, how

**often** did you reach orgasm (climax)?

No sexual activity

Almost always or always

Most times (more than half the time)

Sometimes (about half the time)

A few times (less than half the time) Almost never or never

1. Over the past 4 weeks, when you had sexual stimulation or intercourse, how

**difficult** was it for you to reach orgasm (climax)?

No sexual activity

Extremely difficult or impossible

Very difficult

Difficult Slightly difficult Not difficult

1. Over the past 4 weeks, how **satisfied** were you with your ability to reach orgasm (climax) during sexual activity or intercourse?

No sexual activity

Very satisfied

Moderately satisfied

About equally satisfied and dissatisfied Moderately dissatisfied

Very dissatisfied

1. Over the past 4 weeks, how **satisfied** have you been with the amount of emotional closeness during sexual activity between you and your partner?

No sexual activity

Very satisfied

Moderately satisfied

About equally satisfied and dissatisfied Moderately dissatisfied

Very dissatisfied

1. Over the past 4 weeks, how **satisfied** have you been with your sexual relationship with your partner?

Very satisfied

Moderately satisfied

About equally satisfied and dissatisfied Moderately dissatisfied

Very dissatisfied

1. Over the past 4 weeks, how **satisfied** have you been with your overall sexual life?

Very satisfied

Moderately satisfied

About equally satisfied and dissatisfied Moderately dissatisfied

Very dissatisfied

1. Over the past 4 weeks, how **often** did you experience discomfort or pain during vaginal penetration?

Did not attempt intercourse

Almost always or always

Most times (more than half the time)

Sometimes (about half the time)

A few times (less than half the time)

Almost never or never

1. Over the past 4 weeks, how **often** did you experience discomfort or pain following vaginal penetration?

Did not attempt intercourse

Almost always or always

Most times (more than half the time)

Sometimes (about half the time)

A few times (less than half the time)

Almost never or never

1. Over the past 4 weeks, how would you rate your **level** (degree) of discomfort or pain during or following vaginal penetration?

Did not attempt intercourse

Very high

High

Moderate

Low

Very low or none at all
