# Supplementary material for: Female sexual dysfunction among foreign and Australian-born women: a cross-sectional study
Source: Front Reprod Health. 2026 Jun 16;8:1855626. doi: 10.3389/frph.2026.1855626 (PMC13314969; doi:10.3389/frph.2026.1855626)
Supplement: Supplementary file 2 [file Table2.docx]

**Supplementary Material 2: Supplementary Table 1**

Supplementary Table 1: sensitivity analysis of FSFI domain and total scores among Australian-born and foreign-born women, restricted to responses completed between five and 40 minutes (n = 312)

| **FSFI Domain** Mean ± SD (95% CI) | **Australian-born (n = 123)**  **Mean ± SD [95% CI]** | **Foreign-born (n = 189)**  **Mean ± SD [95% CI]** | **P-value** |
| --- | --- | --- | --- |
| Desire | 3.67 ± 1.27 [3.44, 3.89] | 3.85 ± 1.06 [3.70, 4.01] | 0.162 |
| Arousal | 4.54 ± 1.32 [4.31, 4.78] | 4.58 ± 1.10 [4.42, 4.74] | 0.784 |
| Lubrication | 4.74 ± 1.31 [4.50, 4.97] | 4.77 ± 1.26 [4.59, 4.95] | 0.815 |
| Orgasm | 4.45 ± 1.43 [4.20, 4.71] | 4.33 ± 1.41 [4.13, 4.53] | 0.459 |
| Satisfaction | 4.49 ± 1.19 [4.28, 4.71] | 4.78 ± 1.19 [4.61, 4.95] | 0.039 |
| Pain | 4.94 ± 1.29 [4.71, 5.17] | 4.86 ± 1.34 [4.66, 5.05] | 0.575 |
| FSFI Total Score | 26.84 ± 5.61 [25.83, 27.84] | 27.17 ± 5.59 [26.37, 27.98] | 0.603 |
| FSD, n (%) [95% CI] | 46 (37.4%) [29.4%, 46.2%] | 75 (39.7%) [33.0%, 46.8%] | 0.686 |

Mean ± standard deviation (SD) [95% confidence interval (CI)].

Continuous variables compared using independent samples t-test; categorical variables compared using chi-square test. p values reported to three decimal places.

Higher FSFI domain and total scores indicate better sexual function. FSD defined as an FSFI total score 26.55 or below.

FSFI: Female Sexual Function Index. FSD: Female Sexual Dysfunction.
