# Supplementary material for: Female sexual dysfunction among foreign and Australian-born women: a cross-sectional study
Source: Front Reprod Health. 2026 Jun 16;8:1855626. doi: 10.3389/frph.2026.1855626 (PMC13314969; doi:10.3389/frph.2026.1855626)
Supplement: Supplementary file 3 [file Table3.docx]

Supplementary table 2: Adjusted odds ratios for female sexual dysfunction among Australian women (n = 311)

| **Variable** | **Adjusted OR [95% CI]** |
| --- | --- |
| **Age group** |  |
| 18–29 years | Reference |
| 30–39 years | 1.10 [0.57–2.12] |
| 40–49 years | 1.35 [0.60–3.05] |
| **Education** |  |
| Secondary or less | Reference |
| Bachelor's degree | 0.74 [0.38–1.45] |
| Postgraduate | 0.47 [0.22–1.03] |
| **Marital status** |  |
| Partnered | Reference |
| Single (never married) | 0.98 [0.47–2.06] |
| Previously partnered | 0.20 [0.04–1.06] |
| **Religion** |  |
| No Religion | Reference |
| Atheist | 2.20 [0.68–7.09] |
| Buddhism† | 5.67 [1.35–23.89] |
| Catholic | 0.87 [0.41–1.86] |
| Christian | 1.39 [0.68–2.84] |
| Hinduism | 1.99 [0.59–6.79] |
| Islam | 0.88 [0.28–2.74] |
| Other | 0.83 [0.23–2.97] |
| **IRSAD** |  |
| Most disadvantaged | Reference |
| Disadvantaged | 0.78 [0.22–2.69] |
| Middle | 1.31 [0.45–3.81] |
| Advantaged | 1.38 [0.51–3.76] |
| Most advantaged | 1.38 [0.51–3.77] |
| **Gravidity** |  |
| Nulligravida | Reference |
| One pregnancy | 0.94 [0.45–1.93] |
| Two pregnancies | 0.86 [0.40–1.86] |
| Three or more | 1.11 [0.44–2.80] |
| **Annual household income** |  |
| Low (<$40,000) | Reference |
| Lower-middle ($40,000–$79,999) | 0.49 [0.17–1.38] |
| Upper-middle ($80,000–$149,999) | 0.44 [0.15–1.27] |
| High ($150,000+) | 0.31 [0.10–1.01] |
| **Employment status** |  |
| Employed | Reference |
| Student | 0.67 [0.16–2.74] |
| Unemployed | 0.76 [0.37–1.60] |
| **Visa status** |  |
| Citizen | Reference |
| Work visa | 0.65 [0.23–1.81] |
| Permanent residency | 1.32 [0.67–2.60] |
| Student visa | 1.05 [0.30–3.69] |
| Other | 0.16 [0.02–1.54] |
| **WHO region** |  |
| Western Pacific Region | Reference |
| African Region | 1.70 [0.56–5.13] |
| Region of the Americas | 1.38 [0.46–4.18] |
| South-East Asia Region | 1.47 [0.60–3.60] |
| European Region | 0.86 [0.44–1.69] |
| Eastern Mediterranean Region | 1.39 [0.46–4.22] |

OR = odds ratio; CI = confidence interval; IRSAD = Index of Relative Socio-economic Advantage and Disadvantage; FSD defined as FSFI total score ≤ 26.55. This supplementary model is restricted to overseas-born women only and additionally adjusts for visa status and WHO region of origin, which cannot be included in the combined birthplace model (Table 4). The "Other" WHO region category (n = 4) was omitted due to perfect prediction (zero FSD cases) and those observations were excluded.

The model demonstrated poor fit on the Hosmer-Lemeshow goodness-of-fit test (chi²(8) = 21.56, p = 0.006), likely reflecting the large number of parameters estimated relative to the available sample size. Estimates from this model should therefore be interpreted with caution.
